# Randomized Clinical Trial Comparing the Success Rate of Blockbuster Intubating Laryngeal Mask Airway Versus Fastrach Intubating Laryngeal Mask Airway During Blind Endotracheal Intubation

**DOI:** 10.7759/cureus.39321

**Published:** 2023-05-21

**Authors:** Yuvaraj V, Pratibha SD, Santosh Alalamath, Shivanand L Karigar

**Affiliations:** 1 Anesthesiology, Shri BM Patil Medical College and Hospital, Bijapur Liberal District Education (BLDE) University, Vijayapura, IND

**Keywords:** blind intubation, endotracheal intubation, fiberoptic, intubating laryngeal mask airway, fastrach ilma, blockbuster ilma

## Abstract

Background

A new piece of equipment, the Blockbuster (BB) Intubating Laryngeal Mask Airway (ILMA; Tuoren Medical India, Gurugram, India), was created in 2012. Dr. Chandy created the Fastrach (F) ILMA in 1997, which is another supraglottic airway equipment. The primary purpose of this study was to compare the success rate of intubation with BB-ILMA and F-ILMA.

Methods

In the chosen age category of >20 to <70 years, undergoing general anesthesia for intubation with ILMAs, 55 patients were in the BB-ILMA (group B), and 55 patients were in the F-ILMA (group F). These ILMAs were put in after the induction and checked to see if adequate ventilation was accomplished with either of these devices. Once ventilation had been attained, we proceeded with fiberoptic scope to visualize the glottis, followed by blind intubation. The primary objective was to compare the first pass successful intubation of BB-ILMA and F-ILMA. The secondary objectives were ease of LMA insertion, time taken for intubation, hemodynamic changes, glottis fiberoptic view, and complications.

Results

The first pass successful intubation of the BB-ILMA and F-ILMA are 94.5% and 87.3%, respectively, whereas the time taken for intubation in BB-ILMA and F-ILMA are 25.02 seconds (s) and 42.77 s with a p-value of 0.0001, indicating a statistically significant relationship.

Conclusion

When compared to the F-ILMA (group F), the BB-ILMA (group B) has a higher success rate for blind tracheal intubation, with lesser time taken for intubation and fewer complications.

## Introduction

The most significant effect of general anesthesia on the respiratory system is the diminishing of the airway reflex mechanisms, pharyngeal-laryngeal muscle laxity, hypoventilation, and apnea. Airway management in patients receiving general anesthesia is a set of actions that leads to the development of a safe and secure airway for ventilation. Failure to maintain the airway, which leads to hypoxia, can cause permanent brain damage within minutes [1]. Hence, anesthesiologists must ensure that the airway is secure.

Obesity raises the likelihood of anticipated difficult mask ventilation and failure in tracheal intubation; the chances of oxygen desaturation are very high. Supraglottic airway devices now have a broader range of responsibilities, including airway management in obese and high-risk patients. A supraglottic airway device is used as a safer and easier tracheal intubation during regular procedures or during challenging ones while performing an airway rescue after an unsuccessful intubation attempt. A supraglottic airway device, when used in conjunction with the overall approach, can assist in enhancing the quality of airway management in addition to the patient’s level of safety in obese patients [2].

Supraglottic airway equipment that contains a channel enabling blind tracheal intubation is becoming increasingly popular. It acts as a connection between ventilation and intubation in all patient populations who are undergoing general anesthesia. The Blockbuster Intubating Laryngeal Mask Airway (BB-ILMA) (Tuoren Medical India, Gurugram, India) is a relatively new device that was designed in 2012 in China. It is increasing in popularity since it increases anesthetic safety and quality. It was developed by Professor Ming Tian; benefits include enhanced ventilation and a larger green channel that can be used for intubation [3].

A crucial duty of an anesthesiologist is to maintain the airway. According to the instructions published by the All-India Difficult Airway Association (AIDAA) in 2016, laryngeal mask airways that come equipped with intubation conduits are advantageous and ought to be utilized [4].

Dr. Chandy invented the Fastrach intubating laryngeal mask airway (F-ILMA) around 1997 to serve as a tracheal intubation assistant and guide for patients undergoing general anesthesia. At the moment, F-ILMA is offered in three different sizes: three, four, and five. Each of these sizes can be utilized either once or multiple times [3].

The aim of this study was to compare the success rate of BB-ILMA versus F-ILMA during blind endotracheal intubation. The primary objective was to determine successful intubation on the first attempt. The secondary objectives were ease of laryngeal mask airway (LMA) insertion, time taken for intubation, fiber optic grade of laryngeal view, hemodynamical changes, and complications such as blood staining and sore throat.

## Materials and methods

Sample size

The anticipated success rate of tracheal intubation is 90% in the BB-ILMA group (group B) and 66.6% in the F-ILMA group (group F) [3], respectively; the study would require a sample size of 55 per group. (i.e., a total sample size of 110 patients assuming equal group sizes), to achieve a power of 80% for detecting the difference in proportions between the two groups at a two-sided p-value of 0.05. A p-value of less than 0.05 was considered statistically significant.

Materials and methods

Our present study was a prospective comparative study. This study was carried out in the department of anesthesiology, Shri BM Patil Medical College, Bijapur Liberal District Education (Deemed to be University) Vijayapura, Karnataka. The study period was carried out for a period of one and a half years. Ethical clearance from the Institutional Hospital Ethical Committee (IEC/NO-09/2021) and written informed consent from patients were obtained. Around 110 patients with ages >20 years and <70 years, of both genders and American Society of Anesthesiologists (ASA) grade 1 and 2 who were undergoing elective general anesthesia surgeries were randomly divided into two groups of 55 patients each, group B (BB-ILMA) and group F (F-ILMA), by computerized randomization. 

Inability to give consent for the procedure, local infection of the neck, burns and swellings in the neck region, previous surgeries in the neck, patients with a risk of aspiration, and patients with poor pulmonary compliance were excluded from the study.

**Figure 1:**
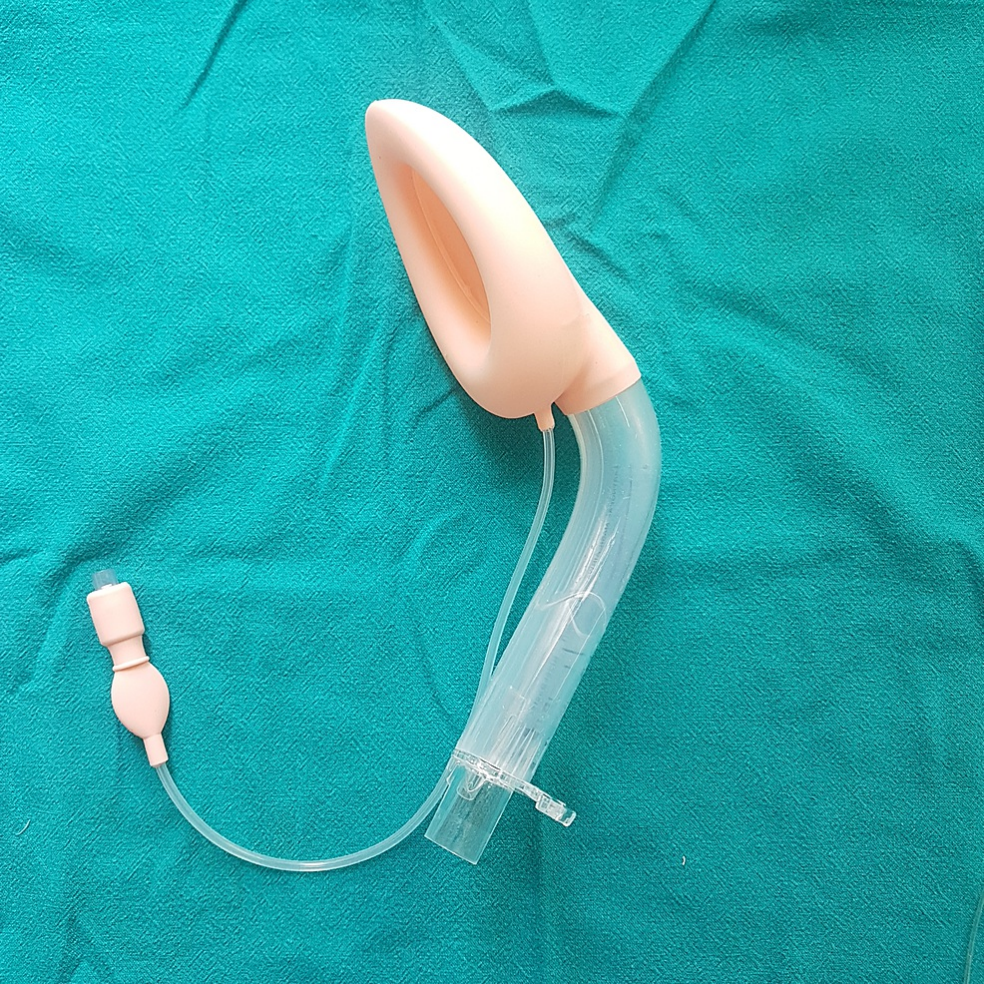
BB-ILMA BB-ILMA - Blockbuster intubating laryngeal mask airway

**Figure 2:**
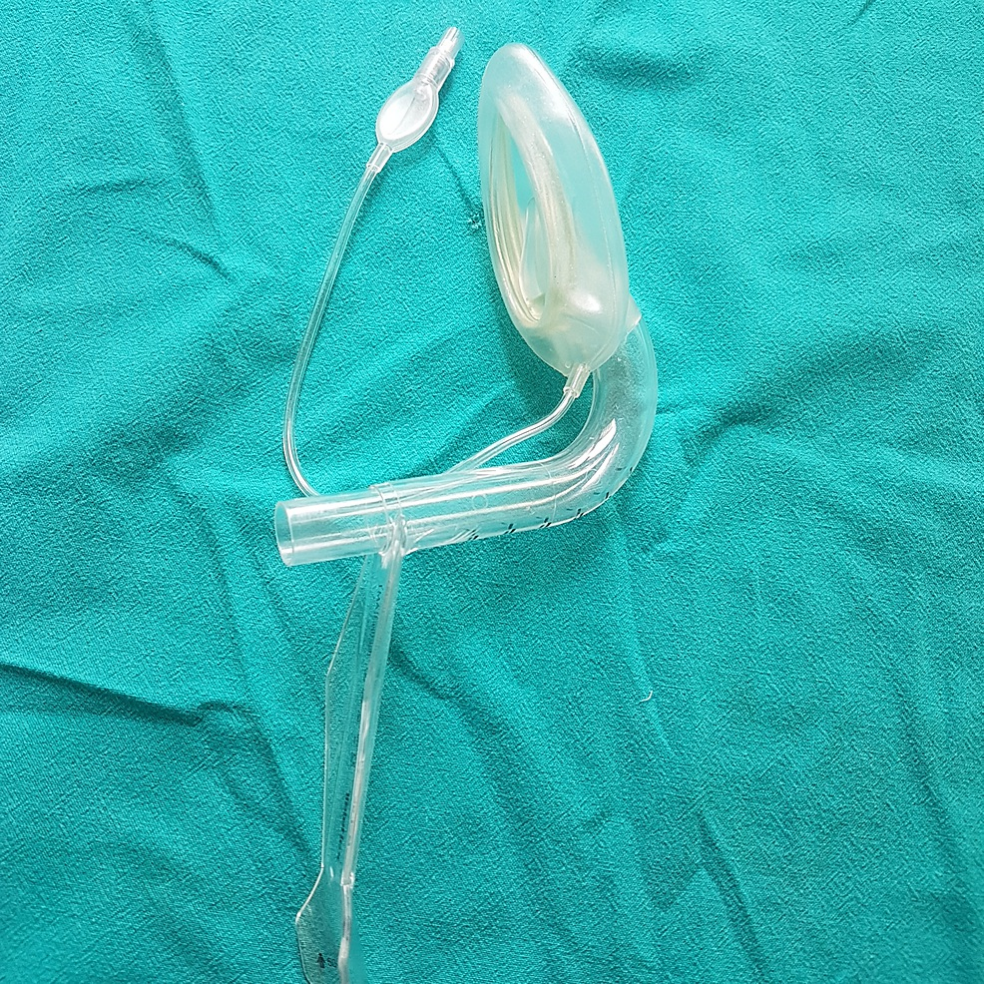
F-ILMA F-ILMA - Fastrach intubating laryngeal mask airway

All patients underwent thorough pre-operative assessment, which includes history of underlying medical illness, previous history of surgery, previous anesthetic exposure, and hospitalization. Physical examination included the general condition of the patient, height, weight, and BMI, vital signs like heart rate, blood pressure, respiratory rate, systemic examination of the cardiovascular system, respiratory system, central nervous system, and airway assessment by Mallampati grading. Patients were randomly allocated into two groups: group B (55 patients) and group F (55 patients). Patients were shifted to the operation theater, and standard monitoring devices were attached, which included a pulse oximeter, non-invasive blood pressure (NIBP), end-tidal carbon dioxide (EtCO2), and electrocardiogram (ECG), and baseline values were recorded. An intravenous line (IV) was secured with an 18G/20G cannula, and all patients were premedicated with injection (inj) ondansetron 0.15mg/kg IV, inj glycopyrrolate 0.008mg/kg IV, inj midazolam 0.08mg/kg IV. Patients were preoxygenated for three to five minutes with 100% oxygen, and inj fentanyl 1mcg/kg IV was given as analgesia. For ILMA insertion, anesthesia was induced with inj propofol 2mg/kg IV, and muscle relaxation was obtained with inj atracurium 0.5mg/kg IV. The individual's body weight was used to determine which size of ILMA was most suitable. According to the recommendations, a size three fits for individuals weighing between 30 to 50 kilograms, while a size four accommodates those weighing between 50 to 70 kilograms, and a size five fits for individuals weighing between 70 to 100 kilograms. The ease of LMA insertion was assessed using a subjective scale of one to four (1 - no resistance, 2 - mild resistance, 3 - moderate resistance, and 4 - inability to place the device). After three minutes, one of the devices was placed using a technique that involves inserting the device midline while the neck is in a neutral position. For BB-ILMA, there was no such technique. To promote BB-ILMA (Figure 1) passage, the patient's mouth was opened, and the tongue was displaced with a disposable sterilized wooden tongue depressor. Pre-lubricated BB-ILMA was inserted, and the cuff was inflated and checked for respiration after connecting the BB-ILMA to the breathing circuit. The Chandy maneuver was carried out on a group of patients who were intubated using an F-ILMA (Figure 2). To promote Fastrach passage, the patient's mouth was opened, and the tongue was displaced with a disposable sterilized wooden tongue depressor. Pre-lubricated F-ILMA was inserted with mild inward and downward pressure, utilizing the device's curvature as a guide, until a set resistance to further progress was sensed, and the cuff was inflated and checked for respiration after connecting the F-ILMA to the breathing circuit. The presence of chest movements and EtCO2 waveforms provided evidence of adequate ventilation.

Fiberoptic view by fiberoptic bronchoscope 3 mm after the ILMA insertion by using Brimacombe score with a grading system which is: 1 - only cords are seen; 2 - cords with posterior epiglottis seen; 3 - cords plus anterior epiglottis seen; 4 - no cords seen. Here grade 1 and 2 indicates for easier insertion of the endotracheal tube (ET). Intubation was done with designed Parker Flex-Tip tubes (Parker Medical, Danbury, Connecticut), which is a type of armored endotracheal tube that is used according to the individuals built, and bilateral air entry was verified. The capacity to obtain a minimum of 7 ml/kg tidal volume using a square wave capnogram served as confirmation of the successful placement of the endotracheal tube. The ease of intubation, the number of attempts, and the time taken for successful intubation were noted.

Statistical analysis

Data were expressed using mean, standard deviation, percentages, and graphs. To compare different groups, the ANOVA test/ Kruskal-Wallis test and post hoc test were utilized. A significant difference between qualitative data was discovered using the chi-square or Fisher's exact test. Data were entered into a Microsoft Excel (Microsoft, Redmond, Washington) spreadsheet for statistical analysis. and the findings were evaluated using SPSS version 27.0 (IBM Inc., Armonk, New York) and GraphPad Prism version 5.

**Figure 3:**
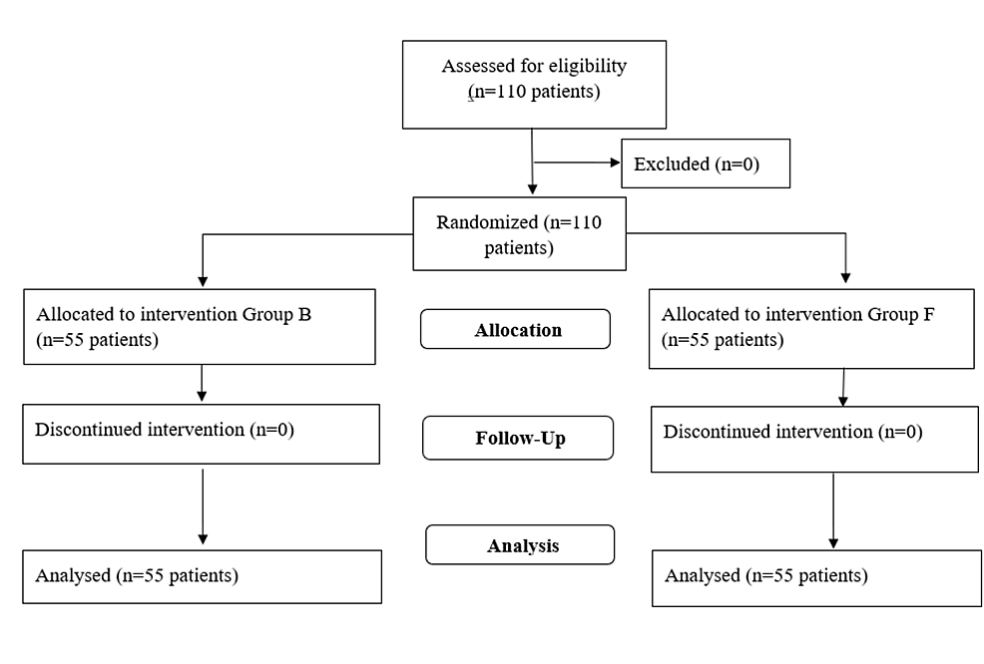
Consort flow chart n - number of patients, group B - Blockbuster intubating laryngeal mask airway group, group F - Fastrach intubating laryngeal mask airway group

Here in the consort flow chart, a sample size of 110 patients is included in the study; they were allocated into two groups for further follow-up and analysis (Figure 3).

## Results

A total of 110 patients were included in the study, who were randomly allocated into two interventional groups - group B and group F with 55 patients in each group. There was no loss to follow-up in either group, and both were analyzed for further results. Demographic variables like age, gender, and ASA status were compared between the two groups (Table 1). 

**Table 1:** Distribution of study variables among the study participants (N=110) SD - standard deviation, ASA - American Society of Anesthesiologists

Variable	Group B	Group F	p-value
Age, years (mean ± SD)	38.56 ± 13.390	38.42 ± 11.229	0.951
Gender			0.4437
Female	27 (49.1%)	23 (41.8%)	
Male	28 (50.9%)	32 (58.2%)	
ASA grade			0.5541
1	33 (60.0%)	36 (65.45%)	
2	22 (40.0%)	19 (34.55%)	

In group B and group F the distribution of mean pulse rate (PR; first min) and mean PR (10th min) was statistically significant (p<0.0001; Table 2). 

**Table 2:** Pulse rate in the first, fifth, and 10th minute PR - pulse rate, SD - standard deviation, min - minute

	Group B		Group F		p-value
	Mean	SD	Mean	SD	
PR (1^st^ min)	83.18	4.252	69.40	4.860	0.0001
PR (5^th^ min)	81.95	4.519	84.24	6.067	0.071
PR (10^th^ min)	82.71	4.593	86.29	5.028	0.0001

In group B and group F, the distribution of mean systolic blood pressure (SBP; first, fifth, 10th min) was not statistically significant (Table 3).

**Table 3:** Systolic blood pressure in the first, fifth, and 10th minute SBP - systolic blood pressure, SD - standard deviation, min - minute

	Group B		Group F		p-value
	Mean	SD	Mean	SD	
SBP (1^st^ min)	117.09	7.619	117.82	6.580	0.542
SBP (5^th^ min)	116.36	7.543	116.73	7.467	0.859
SBP (10^th^ min)	117.09	7.619	115.64	6.876	0.321

In group B and group F, the distribution of mean diastolic blood pressure (DBP; first, fifth, 10th min) was not statistically significant (Table 4).

**Table 4:** Diastolic blood pressure in the first, fifth, and 10th minute DBP - diastolic blood pressure, SD - standard deviation, min - minute

	Group B		Group F		p-value
	Mean	SD	Mean	SD	
DBP (1^st^ min)	77.64	9.019	77.64	9.019	1.000
DBP (5^th^ min)	77.09	9.559	76.36	9.302	0.709
DBP (10^th^ min)	78.55	8.259	77.09	9.364	0.464

Time taken for intubation was more in group F when compared with group B (p-value 0.0001). The first pass successful intubation in group B was more successful than group F, but this was found to be statistically insignificant. The Fiberoptic grading values of group B and group F are statistically significant (Table 5). 

**Table 5:** Variables compared after intubation through LMA devices LMA - laryngeal mask airway

Variables	Group B	Group F	p-value
Ease of LMA insertion			0.184
Easy (scores 1, 2)	52 (94.5%)	48 (87.3%)	
Difficult (scores 3, 4)	3 (5.5%)	7 (12.7%)	
Time taken for intubation in seconds (mean ± SD)	25.02 ± 10.811	42.77 ± 16.289	0.0001
First pass successful intubation			0.184
Successful	52 (94.5%)	48 (87.3%)	
Unsuccessful	3 (5.5%)	7 (12.7%)	
Fiber optic grading (Brimacombe score)			0.0001
Grade 1	26 (47.8%)	48 (87.3%)	
Grade 2	29 (52.7%)	7 (12.7%)	
Complications			0.294
Blood staining	2 (3.6%)	5 (9.1%)	
Sore throat	0 (0%)	1 (1.18%)	
None	53 (96.4%)	49 (89.1%)	

## Discussion

Dr. Ian Archie Jeremy Brain developed the first supraglottic airway equipment in the year 1981 [5]. It plays a significant part in airway management because it enables air to be exchanged through a mask that was designed specifically for the hypopharynx and was positioned such that it accommodates the laryngeal inlet to produce a complete seal.

Blockbuster belongs to a second-generation supraglottic airway device, and it is an anatomically formed airway tube that was placed into the pharynx. This modified supraglottic airway device had been specially created to enable blind or fiberoptic-guided tracheal intubation. The Tourens BB-ILMA was a relatively new intubating laryngeal mask airway (ILMA) that was designed in 2012 in China. It has gained favor as a means of enhancing both the quality and safety of anesthesia [3]. It was introduced by Professor Ming Tian and can be used for ventilation as well as for intubation.

Few studies have been published to assess how successful the ILMA-Fastrach is in the treatment of those who have been diagnosed with difficult airway (DA) [6,7]. The suggested approach is the midline position, with the cuff fully deflated and the mask opening pointing forward. The totally deflated approach was more precise and safer due to the improved fiberoptic vision [8]. F-ILMA was invented in 1997 by Dr. Chandy and it concentrates on intubation. According to studies, the success percentage of blind intubation in both predicted and unexpected airways was roughly 90-95%, with a lower frequency of difficulties developing throughout the process. The Fastrach ILMA (F-ILMA) is designed to serve as an intubation conduit. While other LMA designs can achieve the same effect, the F-ILMA has key distinguishing features that maximize the chances of successful intubation while imposing no size constraints upon that endotracheal tube (ET) [3].

Cricoid pressure was used to reduce the chance of aspiration, although the extent to which it was successful is debatable. It has been demonstrated that applying cricoid pressure reduces the proportion of successful insertions. A surgical airway is necessary and shouldn't be postponed when a patient cannot be intubated or ventilated [9,10]. However, if the LMA is nearby, it can be promptly tried while a helper gets ready for the cricothyroidotomy.

Comparison of demographic variables

Demographic profiles regarding age, gender, and ASA grade in both groups were comparable, and they were statistically not significant (p>0.05).

Endigeri et al. [3], in 2019, used randomization to divide sixty patients aged 20-60 years who were undergoing general anesthesia into two groups of thirty patients each for the goal of achieving tracheal intubation with either a Blockbuster ILMA (group B) or a Fastrach ILMA (group F). They discovered that tracheal intubation success rate was 90% in group B and 66.6% in group F (p=0.3), whereas in our study, the first pass success intubation in group B was 94.5% and in group F was 87.3%. Hence in our study, group B achieved a better success rate on the first attempt of blind tracheal intubation.

Endigeri et al. [3] concluded that the time for intubation (mean ± SD) was 18 ± 3 for group B and 32 ± 4 for group F, with A p-value OF 0.001. In our study, the time taken for intubation in seconds was 25.02 ± 10.811 for group B and 42.77 ± 16.289 for group F, with a p-value of 0.0001, which is statistically significant. The study parameters are comparable.

In our study, the fiber optic grading (Brimacombe score) was done; 26 patients in group B and 48 patients in group F were found to be grade 1, whereas grade 2 was noted in 29 patients of group B and seven patients of group F, with a p-value of 0.0001, which is statistically significant. We have used Chandy's maneuver for endotracheal intubation through F-ILMA. Although a higher number of patients had grade 1 fiber optic view in group F, it was more difficult to intubate because of anatomical changes in the airway due to the angulation of F-ILMA. However, in a study done by Endigeri et al. [3], no such statistical significance was noted.

In contrast to our study, Sinha et al. [11], in 2019, did a study in which they included only obese surgical individuals, whose BMI was more than 35kg/m2, as study subjects. In our study, obese patients were excluded.

Lal et al. [12], in 2020, did a study that consisted of one hundred participants of either gender, ranging in age from 18 to 60 years, belonging to the American Society of Anesthesiologists (ASA) physical status class 1 or 2. With both group A (polyvinyl chloride (PVC) tracheal tube) and group B (Parker Flex-Tip tracheal tube) consisting of 50 patients each, blind intubation was performed using a regular PVC tracheal tube and a Parker Flex-Tip tube, respectively. When compared with traditional PVC tracheal tube, the success rate on the first try for Parker Flex-Tip tracheal tube was statistically higher (p=0.002). In our study, we used a Parker Flex-Tip tracheal tube in both groups B and F; in Group B, it was able to intubate the patients more easily and achieve a higher success rate overall on the very first attempt when compared with Group F.

In the 2005 American Heart Association guidelines for managing patients experiencing cardiac arrest, the LMA was described as a feasible option for intubation [13,14].

Gerstein et al. [15] in their study concluded that F-ILMA has established itself as an effective airway device for usage both in and out of the operating room in cases of airway difficulty. Effective ventilation can be achieved in almost all situations; they were able to perform blind endotracheal intubation in the vast majority of cases. Complications of a serious nature had not been encountered in their study. In our study the complications like blood stains were found in both groups B and F, but there were lesser complications in group B when compared with group F. Hence, group B is better than group F.

From the above discussion of the entire study, group B was found to be superior with respect to the time taken for intubation (seconds) with statistically significant values when compared with group F. 

Limitations of the study

ASA grade three and grade four patients were not included in our study. Obese patients with a BMI>30kg/m^2^ were not included in our study.

## Conclusions

The ease of LMA insertion was better with Blockbuster ILMA. It also had lesser time taken for intubation which is statistically significant. We found that higher first-pass successful intubation was better with BB-ILMA compared to F-ILMA. We also observed that pulse rate variability at the first minute was high with BB-ILMA. Complications like blood stains and sore throats were found to be less with BB-ILMA. F-ILMA had shown positive results only with respect to better fiberoptic view and high pulse rate at the 10th minute. Hence this study encourages the use of BB-ILMA over F-ILMA for successful blind endotracheal intubation. 
